# Multiome-wide Association Studies: Novel Approaches for Understanding Diseases

**DOI:** 10.1093/gpbjnl/qzae077

**Published:** 2024-10-29

**Authors:** Mengting Shao, Kaiyang Chen, Shuting Zhang, Min Tian, Yan Shen, Chen Cao, Ning Gu

**Affiliations:** Key Laboratory for Bio-Electromagnetic Environment and Advanced Medical Theranostics, School of Biomedical Engineering and Informatics, Nanjing Medical University, Nanjing 211166, China; Key Laboratory for Bio-Electromagnetic Environment and Advanced Medical Theranostics, School of Biomedical Engineering and Informatics, Nanjing Medical University, Nanjing 211166, China; Key Laboratory for Bio-Electromagnetic Environment and Advanced Medical Theranostics, School of Biomedical Engineering and Informatics, Nanjing Medical University, Nanjing 211166, China; Key Laboratory for Bio-Electromagnetic Environment and Advanced Medical Theranostics, School of Biomedical Engineering and Informatics, Nanjing Medical University, Nanjing 211166, China; Key Laboratory for Bio-Electromagnetic Environment and Advanced Medical Theranostics, School of Biomedical Engineering and Informatics, Nanjing Medical University, Nanjing 211166, China; Key Laboratory for Bio-Electromagnetic Environment and Advanced Medical Theranostics, School of Biomedical Engineering and Informatics, Nanjing Medical University, Nanjing 211166, China; Key Laboratory for Bio-Electromagnetic Environment and Advanced Medical Theranostics, School of Biomedical Engineering and Informatics, Nanjing Medical University, Nanjing 211166, China; Nanjing Key Laboratory for Cardiovascular Information and Health Engineering Medicine, Institute of Clinical Medicine, Nanjing Drum Tower Hospital, Medical School, Nanjing University, Nanjing 210093, China

**Keywords:** Genome-wide association study, Transcriptome-wide association study, Multiome, Gene-based association study, Complex disease

## Abstract

The rapid development of multiome (transcriptome, proteome, cistrome, imaging, and regulome)-wide association study methods have opened new avenues for biologists to understand the susceptibility genes underlying complex diseases. Thorough comparisons of these methods are essential for selecting the most appropriate tool for a given research objective. This review provides a detailed categorization and summary of the statistical models, use cases, and advantages of recent multiome-wide association studies. In addition, to illustrate gene–disease association studies based on transcriptome-wide association study (TWAS), we collected 478 disease entries across 22 categories from 235 manually reviewed publications. Our analysis reveals that mental disorders are the most frequently studied diseases by TWAS, indicating its potential to deepen our understanding of the genetic architecture of complex diseases. In summary, this review underscores the importance of multiome-wide association studies in elucidating complex diseases and highlights the significance of selecting the appropriate method for each study.

## Introduction

The genome-wide association study (GWAS) was first proposed in 2005 [1] and aim to discover statistical associations between millions of genomic variants and phenotypes, particularly for complex traits in humans [2–6]. GWAS screens a large number of whole genomes to find variants that appear more frequently in individuals with a specific trait than those without it. Over the past two decades, research into GWAS methods has expanded rapidly, and GWAS has been successfully applied in many datasets to identify numerous genetic variants related to multiple complex traits and diseases [7–9]. However, GWAS still has significant limitations such as poor interpretability and insufficient statistical power [10], due to its use of statistical associations to estimate the correlation, not causation, between variants and traits. Furthermore, many false-positive associations have been identified by GWAS as a result of linkage disequilibrium (LD) [11–13].

**Figure 1:**
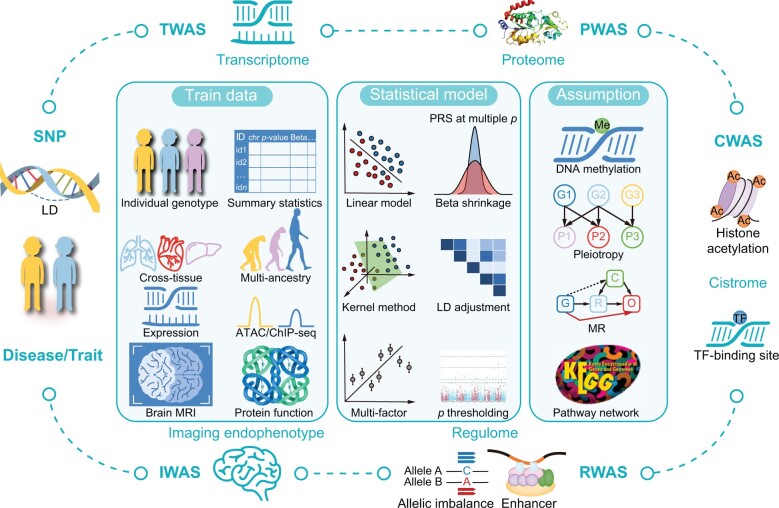
Overview of multiome-wide association study methods This figure depicts the datasets, models, and biological assumptions employed in the development of multiome-wide association study methods. Outer ring: path indicating how multiome-wide association study methods link SNPs to diseases or traits, thereby increasing the interpretation and power of traditional GWAS methods. Left: from top to bottom, three parts show primary data sources used in multiome-wide association study methods. Starting with individual-level genotype data and GWAS summary statistics. Then some extensions to TWAS methods distinguish between various tissues (cross-tissue) or ancestries (multi-ancestry) to search for tissue- or ancestry-dependent variants and mechanisms. Finally, data sources for five multiome-mediated methods are also shown, which are expression data for TWAS, ATAC-seq data for RWAS, ChIP-seq data for CWAS, imaging data for IWAS, and protein function data for PWAS. Middle: statistical models used in multiome-mediated methods. Linear models such as LASSO and BSLMM are widely used in the traditional pipeline, and the kernel machine method is a non-linear model for capturing the interaction of variants. The multi-factor model is used to investigate the effects of multiple genes on traits. Beta shrinkage, LD adjustment, and multiple *p*-value thresholding are widely applied in polygenic risk score methods, which extend TWAS methods to enable the training of models on summary statistics. Right: biological assumptions used in multiome-mediated methods. Epigenetic data, such as DNA methylation, are based on the assumption that integrated *cis*-regulatory elements can help improve the accuracy of expression prediction. Pleiotropy, MR, and pathway networks are other common tools used to enhance the power of multiome-mediated methods. P, phenotype; C, confounding factor; O, outcome; R, risk factor; G, genetic variant; SNP, single-nucleotide polymorphism; GWAS, genome-wide association study; TWAS, transcriptome-wide association study; PWAS, proteome-wide association study; CWAS, cistrome-wide association study; RWAS, regulome-wide association study; IWAS, imaging-wide association study; LD, linkage disequilibrium; ATAC-seq, assay for transposase-accessible chromatin using sequencing; ChIP-seq, chromatin immunoprecipitation followed by sequencing; MRI, magnetic resonance imaging; PRS, polygenic risk score; TF, transcription factor; Me, methylation; Ac, acetylation; LASSO, least absolute shrinkage and selection operator; BSLMM, Bayesian sparse linear mixed model; MR, Mendelian randomization.

**Figure 2:**
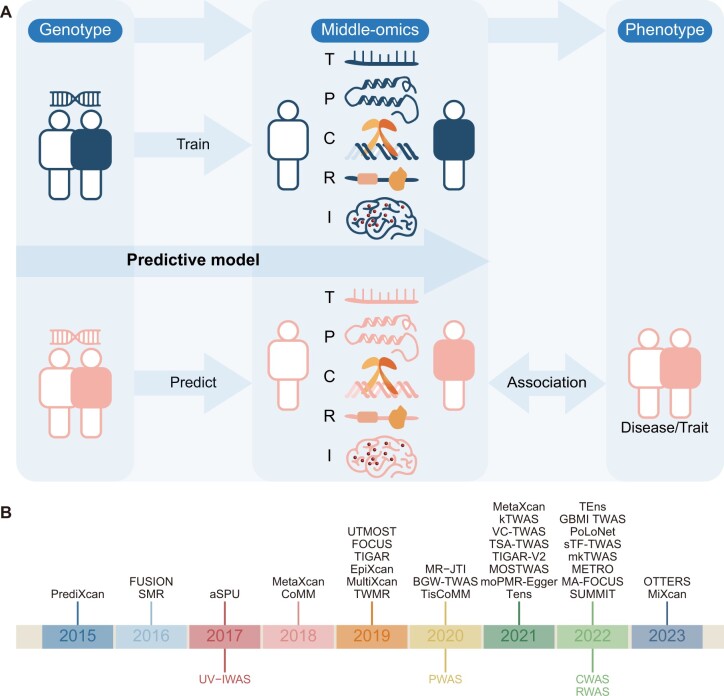
General framework and landmark publications of multiome-wide association study methods **A**. The general workflow of multiome-wide association study methods. First (top), different statistical models are utilized to estimate how genotype determines multiome-mediated features such as the transcriptome (T), proteome (P), cistrome (C), regulome (R), or images (I). Then, the trained model is used to predict multiome-mediated features from test data. Finally, associations between the predicted multiome-mediated features and complex human traits or diseases are identified (bottom). **B**. Timeline for the development of key multiome-wide association study methods over the past few years. The upper black font represents the TWAS methods, while non-TWAS methods are indicated by colored font below the year.

With the development of high-throughput sequencing technologies and the increasing number of multiomic datasets, such as transcriptomes, proteomes, and epigenomes, many multiome-wide association study methods have emerged to address the limitations of GWAS. These innovative methods include transcriptome-wide association study (TWAS) [14,15], proteome-wide association study (PWAS) [16], cistrome-wide association study (CWAS) [17], imaging-wide association study (IWAS) [18–20], and regulome-wide association study (RWAS) [21,22] (**Figure 1**). These approaches are collectively termed “multiome-mediated” methods due to their use of an additional data source as an intermediate step between genotype and phenotype association (**Figure 2**A). Generally, multiome-mediated methods aim to identify biomarkers related to both genetic variants and phenotypes, thereby improving statistical power and enabling the interpretation of genetic variants through their associated biomarkers (Figure 2A). The integrated analysis of these multiomic datasets refines our understanding of human traits by providing insights into the genetic determinants underlying complex diseases.

TWAS was the first framework established to integrate multiomic data following the development of GWAS (Figures 1 and 2B). The two earliest TWAS methodologies PrediXcan [14] and TWAS-FUSION [15] have been widely adopted, allowing biologists to better identify and interpret susceptibility genes in various diseases [23–27], such as calcific aortic valve stenosis [28], macular degeneration [29], schizophrenia [30,31], and also applied into other domains, including drug repurposing [32,33].

Following the development of TWAS, the proteome was next integrated with GWAS, based on the hypothesis that genetic variants in coding regions influence phenotypes by affecting the biochemical function of protein products. PWAS, introduced in 2020 [16] (Figures 1 and 2B), is a novel framework for identifying gene–trait associations mediated by functional changes in proteins. PWAS estimates the effect of variants on protein function using FIRM [34], a machine-learning model that considers the proteomic context of each variant. This approach addresses the limitations of GWAS, particularly the poor interpretability and limited statistical power due to numerous variant loci.

Similar to PWAS, IWAS was proposed in 2017 [18] (Figures 1 and 2B). This approach is a potent method that integrates GWAS with imaging endophenotypes. It has been demonstrated that IWAS enhances statistical power and improves the biological interpretability of GWAS findings [35]. Additionally, IWAS has shown that the TWAS methodology can be extended to a larger variety of endophenotypes beyond gene expression, with many potential applications.

Recently, CWAS [17] was introduced to integrate GWAS with epigenomics (Figures 1 and 2B). The main goal of CWAS is to discover chromatin quantitative trait loci (cQTLs) and discern allele-specific effects on chromatin states associated with traits. The efficacy of CWAS has been demonstrated through its application to epigenetic and GWAS summary statistic data on prostate cancer. In contrast to utilizing expression quantitative trait loci (eQTLs) in TWAS, CWAS can detect and prioritize more variants relative to transcriptome factors, making it a promising approach for future genetic investigations.

Additionally, two independent methods termed RWAS have recently been published [21,22] (Figures 1 and 2B). The first method employs stratAS [36] for allelic imbalance analysis using cancer assay for transposase-accessible chromatin using sequencing (ATAC-seq) data. It then identifies associated regulatory elements, their impact mechanism, and potential upstream regulators through allele-specific accessibility QTLs (as-aQTLs). Another pipeline, facilitated by the MAGMA software package [37], aims to link enhancers with diseases and has shown success applied to schizophrenia data. Essentially, these two RWAS pipelines represent innovative approaches that integrate regulome data with GWAS data. They hold promise for improving the power and interpretability of GWAS findings, thereby aiding in discovering novel genetic factors underlying complex traits.

As this brief history of multiome-mediated methods demonstrates, there are many different ways to improve and modify existing GWAS methods that have been successfully used to identify disease-associated genes. To aid researchers navigate the large number of methods that have been developed, our review provides a comprehensive summary and analysis of the statistical models, use cases, and advantages of all currently available multiome-wide association study methods. Furthermore, using Alzheimer’s disease (AD) as an example, we discussed multiple studies employing various TWAS methods to discover susceptibility genes associated with AD. We also explored the limitations of current studies as well as the factors impacting the association results.

## Classification of multiome-wide association study methods

### TWAS

TWAS, a gene-based approach, identifies associations between genetically regulated expression (GReX) components and complex diseases or traits. The biological hypothesis underlying GReX is that variations in complex diseases and traits often result from the cumulative effect of genetic variations across many genes, with single genetic variants typically exerting small effects on phenotypic variation [38,39]. Thus, TWAS considers the collective contribution of single-nucleotide polymorphism (SNPs) within a single gene or genetic region to phenotype rather than focusing on a single locus of genetic variation. Notably, eQTL represents a crucial class of regulatory variations elucidating the relationship between genetic variants and the regulation of transcription and translation [40–42].

The traditional TWAS framework comprises three steps (Figure 2A). First, an imputation model, such as a linear model, is trained on a reference panel (*e.g.*, Genotype-Tissue Expression Consortium [43]) containing matched genotype and transcriptome data. This model utilizes individual-level genotypes to predict gene expression associated with GReX [14]. Subsequently, a single-tissue or cross-tissue model is applied to a GWAS cohort lacking transcriptome data to estimate gene expression from the genetic variants within the cohort [44,45]. Finally, gene–trait association tests are conducted on the predicted expression, either within a specific tissue or across multiple tissues.

TWAS stands out as the most widely used multiome-wide association study. Since its inception in 2015 [14], various improvements have been made to the traditional TWAS approach to boost accuracy and applicability. Here, we review representative extension methods, categorizing them into five distinct groups based on the differences in algorithmic construction and statistical models employed in various TWASs (**Table 1**).

**Table 1:** Classification and detailed information of the multiome-wide association study methods

Classification		Method	Website	Description	Input	Output	Data	Ref.
TWAS	Improving GReX accuracy	PrediXcan	https://github.com/hakyimlab/PrediXcan	Correlate estimated genetically regulated gene expression with the phenotype	IGD	GTAs	GTEx; GEUVADIS; DGN; WTCCC; BioVU	[14]
		FUSION	http://gusevlab.org/projects/fusion/	Train weights between *cis*-SNPs and gene expression based on BSLMM	IGD; GSS	GTAs	METSIM; YFS; NTR; lipids2010; MuTHER	[15]
		TIGAR	https://github.com/yanglab-emory/TIGAR	Obtain the weights between genes based on a nonparametric Bayesian model	IGD; GSS	GTAs	ROS/MAP; 1000 Genomes Project; IGAP	[50]
		TIGAR-V2	https://github.com/yanglab-emory/TIGAR	Take VCF files as input and provide both DPR and elastic net to train imputation models	IGD; GSS	GTAs	GTEx; BCAC; OCAC	[51]
		FOCUS	https://github.com/bogdanlab/focus/	Calculate the probability of each gene associated with the TWAS signal based on Bayesian model	GSS	GTAs	1000 Genomes Project; lipids2010	[58]
		MA-FOCUS	https://github.com/mancusolab/ma-focus	Identify causal genes by considering which are shared across ancestries related to diseases or complex traits	GSS	GTAs	GENOA; GEUVADIS; EAS; HapMap; DisGeNET	[59]
		TSA-TWAS	https://github.com/RitchieLab/multi_tissue_twas_sim	Identify tissue-specific causal genes	IGD; GSS	GTAs	ACTG; GTEx	[44]
	Adopting cross-tissue datasets	UTMOST	https://github.com/Joker-Jerome/UTMOST/	Apply cross-tissue weights on expression imputation	IGD; GSS	GTAs	GERA; IGAP; 1000 Genomes Project; GTEx; GEUVADIS	[45]
		MultiXcan/S-MultiXcan	https://github.com/ hakyimlab/MetaXcan	Utilize the first *k* principal components of the estimated gene expression in multiple tissues	IGD; GSS	GTAs	GTEx; UKBB; EGA	[61]
		MR-JTI	https://github.com/gamazonlab/MR-JTI	Combine MR and TWAS	IGD; GSS	GTAs	GTEx; PsychENCODE; ENCODE; Roadmap; UKBB; GEUVADIS	[62]
		TisCoMM/TisCoMM-S	https://github.com/XingjieShi/TisCoMM	Identify the tissue-specific gene–trait associations	IGD; GSS	GTAs	GTEx; NFBC1966; UKBB; 1000 Genomes Project	[63]
	Separating feature selection and aggregation	kTWAS	https://github.com/theLongLab/kTWAS	Integrate kernel-machine with TWAS	IGD	GTAs	1000 Genomes Project; GTEx; WTCCC; MSSNG	[65]
		VC-TWAS	https://github.com/yanglab-emory/VC_TWAS	Assume the effects of *cis*-eQTL SNPs on phenotype are random and use an equivalent kernel test for aggregation	IGD; GSS	GTAs	ROS/MAP; 1000 Genomes Project; MCADGS; IGAP; Synapse	[66]
		mkTWAS	https://github.com/theLongLab/mkTWAS	Replace GReX with marginal effect-based feature selection and kernel-based feature aggregation	IGD	GTAs	1000 Genomes Project; GTEx; WTCCC; DisGeNET	[71]
	Integrating other biological information	EpiXcan	https://bitbucket.org/roussoslab/epixcan	Integrate epigenome to estimate the functional importance of genetic variations on gene expression	GSS	GTAs	CMC; GTEx; STARNET; REMC; DLPFC; MSigDB; Harmonizome	[72]
		aSPU	https://github.com/ChongWu-Biostat/aSPU2	Conduct association test by integrating single set or multiple sets of weights	IGD; GSS	GTAs	WTCCC; lipids2010; lipids2013; HapMap; DGN; 1000 Genomes Project; NTR; YFS; METSIM	[74]
		BGW-TWAS	https://github.com/yanglab-emory/BGW-TWAS	Integrate both *cis*- and *trans*-eQTL weights to predict expression	IGD; IGWD	GTAs	ROS/MAP; MCADGS; RADC; 1000 Genomes Project; IGAP	[67]
		MOSTWAS	https://github.com/bhattacharya-a-bt/MOSTWAS	Prioritize distal-SNPs contributing to genes	IGD; IGWD	GTAs	ROS/MAP; TCGA; PsychENCODE; IGAP; PGC; UKBB; iCOGs	[76]
		METRO	https://github.com/zhengli09/METRO	Utilize expression data from multiple ancestries	IGD; GSS	GTAs	GENOA; GEUVADIS; UKBB; GLGC; AAAGC; MEDIA; 1000 Genomes project; GENCODE	[81]
		GBMI-TWAS	https://github.com/bhattacharya-a-bt/gbmi_twas	Conduct inverse-variance weighted meta-analysis on multi-ancestry datasets from GBMI	GSS	GTAs	1000 Genomes project; GBMI; UKBB	[85]
		moPMR-Egger	https://github.com/yuanzhongshang/PMR	Investigate causal influence on multiple traits based on LD between *cis*-SNPs of one gene	IGD; GSS	GTAs	GEUVADIS; GERA; UKBB	[95]
		TEns	https://zenodo.org/record/6845955	Identify eQTLs of transcribed enhancers	GSS	GTAs	Synapse; SuperAgerEpiMap; GTEx	[98]
		sTF-TWAS	https://github.com/theLongLab/TF-TWAS	Integrate prior knowledge of susceptible TF occupied elements to TWAS	IGD; GSS	GTAs	GTEx; BCAC; PGC; PRACTICAL; TRICL-ILCCO; LC3	[101]
		PoLoNet	https://github.com/Liye222/PoLoNet	Integrate biological network regression and proportional odds logistic model in TWAS	IGD	GETAs	GEUVADIS; UKBB; KEGG; GCTA; GENCODE	[103]
	Training with summary statistics	OTTERS	https://github.com/daiqile96/OTTERS	Adopt multiple PRS methods to estimate eQTL weights	GSS	GTAs	UKBB; GTEx; eQTLGen; 1000 Genomes Project; ROS/MAP; MSBB; UKBB	[106]
		Multi-variant TWAS	/	Utilize multi-variant TWAS models and larger eQTL summary statistic datasets to identify causal genes	ESS	GTAs	GTEx; eQTLGen; MetaBrain; YFS; GTEx; HapMap3	[110]
PWAS		/	https://github.com/nadavbra/pwas	Aggregate the signal of all variants jointly affecting a protein-coding gene and assess their overall impact on the protein’s function	IGD	PGTAs	UKBB	[16]
CWAS		/	https://github.com/scbaca/cwas	Identify chromatin (cistrome) features that are genetically associated with a trait of interest	GSS	CTAs	GSE205885; GTEx; 1000 Genomes Project	[17]
RWAS		/	https://zenodo.org/record/6371678	Consider as-aQTLs’ influence on disease risk	GSS	RTAs	TCGA; GTEx; ENCODE	[21]
			https://github.com/casalex/RWAS	Identify the characteristics of enhancers that contribute to genetic risk for disease	GSS	ETAs	GTEx; Roadmap; Hi-C data; PGC; UCSC	[22]
IWAS		UV-IWAS	https://github.com/kathalexknuts/MVIWAS	Exchange transcriptome by using imaging endophenotypes	IGWD; GSS	GTAs	ADNI; GTEx; IGAP; lipids2013	[18]
		MV-IWAS	https://github.com/kathalexknuts/MVIWAS	Exchange transcriptome by using imaging and other endophenotypes	IGWD; GSS	GTAs	ADNI; UKBB; ENIGMA; IGAP	[19]
		BrainXcan	https://github.com/hakyimlab/brainxcan	Leverage reference brain MRI data	IGD; GSS	BTAs	UKBB; HapMap3; PGC	[20]

*Note*: GWAS, genome-wide association study; TWAS, transcriptome-wide association study; PWAS, proteome-wide association study; CWAS, cistrome-wide association study; RWAS, regulome-wide association study; IWAS, imaging-wide association study; SNP, single-nucleotide polymorphism; BSLMM, Bayesian sparse linear mixed model; VCF, variant call format; DPR, Dirichlet process regression; GReX, genetically regulated expression; PRS, polygenic risk score; MRI, magnetic resonance imaging; IGD, individual-level genotype data; GSS, GWAS summary statistics; IGWD, individual-level GWAS data; eQTL, expression quantitative trait locus; as-aQTL, allele-specific accessibility quantitative trait locus; MR, Mendelian randomization; LD, linkage disequilibrium; TF, transcription factor; ESS, eQTL summary statistics; GTA, gene–trait association; GETA, gene/edge–trait association; PGTA, protein-coding gene–trait association; CTA, cistrome–trait association; RTA, regulome–trait association; ETA, enhancer–trait association; BTA, brain feature–trait association. The abbreviations of all databases are detailed in Table S3.

#### Extension in improving GReX accuracy

The first category focuses on improving the accuracy of GReX predictions. GReX, initially proposed in 2015 with the development of PrediXcan [14], focuses on the mechanism of gene expression regulation, excluding the effect of environmental factors and traits. PrediXcan employs the elastic net [46] to train weights between *cis*-SNPs and gene expression based on a reference transcriptome dataset. These weights, along with individual-level genotype data, are utilized to predict GReX, which is then assessed for association with phenotypes of interest.

Subsequently, a Bayesian sparse linear mixed model (BSLMM)-based method, FUSION [15] was presented, which achieves the best power in the BSLMM model among five statistical models also including best linear unbiased predictor (BLUP), least absolute shrinkage and selection operator (LASSO) regression model [47], elastic net [46], and the most significantly associated SNP model. FUSION uses BSLMM to capture weights between *cis*-SNPs and gene expression, and this model [48] combines the Bayesian variable selection regression (BVSR) model and the linear mixed model (LMM), offering a higher degree of accuracy. The traditional TWAS framework is derived from PrediXcan and FUSION. Although the two methods differ in their training models and input data types, they form the basis of the standard TWAS approach. Researchers have continued to refine each step of the traditional framework, resulting in extensive research [49].

Compared with parametric imputation methods adopted by both PrediXcan and FUSION, the nonparametric Bayesian model is also suitable for estimating the complex effect of genetics on the transcriptome because it takes parametric models as special cases. Thus, a nonparametric Bayesian method was proposed to estimate the effects of *cis*-eQTLs through data-driven approaches [50]. Comparative analysis indicated that the nonparametric Bayesian method provided a better fit for transcriptomic imputation models under specific settings than the parametric Bayesian method. The authors also introduced TIGAR [50], a software tool that allows for the use of both parametric and nonparametric methods.

To increase efficiency and reduce computational costs, TIGAR-V2 [51] was developed. It directly accepts variant call format (VCF) files of individual-level and summary-level GWAS data as input, and enables parallel computation. TIGAR-V2 offers nonparametric Bayesian Dirichlet process regression (DPR) [52] and elastic net for training imputation models. TIGAR-V2 reduces training computation time by 90% and memory usage by 50% compared with TIGAR [50].

Beyond the model construction step of TWAS, confounding factors such as LD and co-regulation in the genome and transcriptome may reduce the accuracy of TWAS [41,53–57]. FOCUS [58] was proposed to address this. It utilizes a standard Bayesian approach [48] to compute the posterior inclusion probability of each gene associated with the TWAS signal, effectively controlling the effects of LD, predictive weights, and pleiotropy on association outcomes. To avoid overfitting, FOCUS employs a multivariate Gaussian prior, which leads to strong agreement between simulation results and real data. To improve the identification power of susceptibility genes, the multi-ancestry FOCUS model (MA-FOCUS) [59] was developed in 2022, assuming that disease or complex trait-related genes are shared across ancestries [60]. MA-FOCUS employs a Bayesian approach for posterior computation, and simulation results demonstrate its robustness across various datasets and its efficacy in identifying susceptibility genes across ancestries.

While the aforementioned TWAS methods were designed under distinct biological assumptions regarding gene contribution to complex traits or *cis*-SNP effects on gene expression, their efficacy under different biological scenarios remains unclear. Thus, a novel TWAS method, tissue specificity-aware TWAS (TSA-TWAS) [44], was developed to capture the effect of tissue specificities on TWAS power. In simulation studies, TSA-TWAS utilizes two representative prediction models, elastic net and LASSO, and two association approaches, principal component (PC) regression [61] and generalized Berk-Jones (GBJ) test [45], to show that different TWAS protocols have different power based on different biological questions. TSA-TWAS integrates and maximizes the power of multiple methods while controlling type-I error rates based on the single tissue matched to the trait-related tissue. Applying TSA-TWAS to acquired immune deficiency syndrome (AIDS) Clinical Trial Group (ACTG) clinical trial data highlighted its ability to identify both statistically significant and novel associations.

#### Extension in adopting cross-tissue datasets

To overcome the limitation of single-tissue sample size, UTMOST [45] was proposed to perform cross-tissue gene expression imputation using multivariate regression. It improves the prediction accuracy across all available tissues by combining the results of testing used in each tissue with the cross-tissue z-score accuracy of expression values. Another method, MultiXcan [61], also utilizes multivariate regression for gene–trait association testing across multiple tissues but differs from UTMOST by utilizing the first *k* PCs of the predicted gene expression. Real data results applied on 222 traits in UK Biobank (UKBB) demonstrate that MultiXcan can discover more associations for some traits, while simulation results reveal that MultiXcan performs poorly under the assumption that causal expression in a specific tissue is known.

In addition to cross-tissue approaches, the Mendelian randomization (MR) method helps avoid reverse causation and detect confounding factors in observational studies by examining the causal effect of exposure variables on phenotypes. To further improve TWAS accuracy, MR-JTI (joint-tissue imputation) method [62] was proposed, utilizing tissue similarities in gene transcriptome and epigenome data as weights between different tissues. On the other hand, to ensure tissue-specific gene–trait associations, TisCoMM [63] was proposed to demonstrate the co-regulation of genetic variations across different tissues using a unified probabilistic model, achieving robust results with a low false-positive rate.

These improvements have significant implications for identifying the associations between genetic variants and diseases, overcoming dataset sample size limitations and furthering our understanding of underlying mechanisms and potential treatment targets.

#### Extension in separating feature selection and aggregation

Traditional two-stage TWAS methods first build an expression imputation model using genotype and reference transcriptomic data, and then conduct an association test between GReX and the phenotype. These tools assume that the effect of genetic variants on reference transcriptomic data and phenotype is linear. Consequently, GReX is derived as a linear combination of genetic variants, and the same linear model links GReX and phenotype. However, various factors can influence the power of TWAS methods, such as population differences between modeling and test datasets and the non-linear effect of variants on phenotype [64].

Recently, several new kernel-based methods have been developed to improve TWAS power. One such protocol is kernel-based TWAS (kTWAS) [65], which integrates the TWAS-like method and the sequence kernel association test (SKAT)-like method for feature selection and aggregation, separately. Extensive simulations have shown that kTWAS performs robustly and is more resistant to non-linear effects by combining the advantages of both methods. Especially, it can also detect interactions between numerous variants.

Another method to improve TWAS power is the variance-component TWAS (VC-TWAS) method [66], which uses a random effect model suitable for both individual-level and summary-level GWAS data, making it effective for analyzing continuous and dichotomous phenotypes. VC-TWAS estimates eQTL effect sizes using various methods like elastic net [14], nonparametric Bayesian DPR [50], and BVSR [67]. It then adopts a powerful framework analogous to SKAT [68–70] method to factorize the kernel matrix. Simulation tests have shown that VC-TWAS achieves greater power when the linearity assumption is relaxed.

Another promising extension of TWAS is the marginal + kernel TWAS (mkTWAS) [71], which utilizes marginal effect-based and kernel-based methods for feature selection and aggregation to replace GReX. Analyses of real and simulated data have demonstrated that mkTWAS significantly increases the power of identifying susceptibility genes by applying feature selection and aggregation into different statistical models.

These novel TWAS protocols set themselves apart from traditional two-stage methods by decoupling feature selection and aggregation, resulting in improved power. They offer a viable alternative to GReX in most scenarios.

#### Extension in integrating other biological information

In addition to three modifications mentioned above to the traditional TWAS approaches, integrating biological information such as pathway networks and epigenetic data is also a promising strategy for improving gene expression imputation accuracy.

EpiXcan [72], based on the assumption that SNPs within *cis*-regulatory elements (CREs) are more likely to influence gene expression regulation and exhibit functional relevance [73], has improved the accuracy of transcriptome imputation. By incorporating epigenetic data, including DNA methylation, histone modification, and chromatin accessibility, EpiXcan improves the prediction of the functional effect of genetic variations on gene expression levels.

Alternatively, another way to extend the traditional TWAS frameworks is to improve the association testing methodology between genes and traits. To achieve this goal, an adaptive sum of powered score (aSPU) [74] test was proposed based on generalized linear models (GLMs). This approach integrates multiple sets of weights obtained from diverse datasets to conduct association tests, thus it is capable of uncovering more novel gene–trait associations.

Numerous TWAS frameworks are limited to considering only *cis*-eQTLs, neglecting *trans*-eQTLs due to the computational burden. However, emerging research indicates that *trans*-eQTLs are crucial in explaining the expression of most genes [75]. To address this, Bayesian genome-wide TWAS (BGW-TWAS) [67] was proposed to enable the accounting of both *cis*-eQTL and *trans*-eQTL weights, utilizing summary statistics from standard eQTL analysis, allowing for effective computation through Bayesian variable selection regression.

To account for the effect of distal SNPs and other biomarkers underlying the SNP–gene associations, the Multi-Omic Strategies for TWAS (MOSTWAS) [76] method was developed. MOSTWAS integrates distal-SNPs and multiomic data, including epigenomes, microRNAs, and transcription factors (TFs), for transcriptome imputation. This approach enables more robust testing of gene–trait associations, achieving greater power in the process.

Allele frequency and LD patterns differ across populations with diverse genetic backgrounds, which can significantly impact eQTL analysis results and the identification of disease susceptibility genes [77–80]. To address this, METRO [81] was developed, it utilizes expression data from multiple ancestries to improve statistical power through a joint likelihood-based inference model. Additionally, METRO enables the inference of how distinct genetic ancestries’ expression prediction models contribute to explaining gene–trait associations. Probing the ancestry-dependent transcriptome mechanisms driving gene–trait associations enables gaining deeper insights into the genetic underpinnings of complex traits.

Biobanks have been crucial in identifying relationships between genetic variants and human traits, but they have historically comprised primarily individuals of European descent [82,83]. This lack of diversity can influence genetic discoveries and limit our understanding of complex diseases in non-European populations. Different ancestry groups may have varying frequencies of disease-associated genetic variants, highlighting the need for more diverse biobanks to improve our understanding of complex diseases across all populations.

The Global Biobank Meta-analysis Initiative (GBMI) [84] aims to address this issue by fostering collaboration among over 20 biobank resources worldwide. Recently, a pipeline called GBMI-TWAS [85] has been introduced. This pipeline utilizes inverse-variance weighted meta-analysis on multi-ancestry datasets derived from GBMI. It describes practical considerations related to ancestry and tissue specificities, meta-analytic techniques, and challenges encountered at each stage of the TWAS framework. For instance, a comparison between ancestry-aligned and ancestry-misaligned models revealed that utilizing training and testing samples of the same ancestry enhances the predictive capability of the model during expression prediction model construction. The effectiveness of TWAS is influenced by both the expression prediction model and expression heritability [10,86]. Consequently, this finding holds significance in selecting a reference expression panel for actual data analysis. Moreover, another crucial result highlights the importance of employing a suitable ancestry LD reference panel in the meta-analysis of GWAS summary statistics to ensure the accuracy of TWAS associations. By implementing ancestry-specific expression models that adhere to the ancestry-stratified TWAS framework, GBMI-TWAS demonstrates minimal test statistic inflation. These findings underscore the impact of ancestry-specific genetic architecture on gene–trait associations in disease studies and offer insights into the influence of genetic variants on disease susceptibility in populations of diverse ancestries.

Integrating MR to identify gene–trait associations has emerged as a growing trend. In contrast to the traditional two-stage TWAS approach, SMR method [41] utilizes the two-step least-squares (2SLS) method to detect gene–trait associations based on GWAS summary and eQTL data. Additionally, the method incorporates heterogeneity in dependent instruments (HEIDI) approach to identify potential pleiotropic effects of genes. Building upon this framework, TWMR approach [87] combines MR with TWAS to pinpoint causal gene–trait associations. This method utilizes multiple SNPs and gene expression data as instrumental variables and exposures, respectively, thereby mitigating biases arising from pleiotropic effects.

Due to the limited availability of paired genotype and single-cell transcriptome data, most TWAS approaches identify gene–trait associations using bulk transcriptome data. In contrast, MiXcan [88] has been developed as a framework for conducting cell type-level TWAS, enabling the identification of disease-associated cell types and genes. By leveraging prior information on scaled xCell [89] cell type enrichment scores from the training data, MiXcan enhances the estimation of cell type proportions through fitting mixture models for the expression levels of cell type signature genes. However, the current MiXcan framework only decomposes a tissue into two cell type components, necessitating an extension to create a comprehensive TWAS model encompassing all cell types.

SUMMIT [90] is a TWAS method designed to predict gene expression using eQTL summary-level data. Subsequently, associations between the predicted gene expression levels and traits are tested for the selected fitted expression prediction model. The combined *p*-value is then aggregated using the Cauchy combination test [91,92] to quantify the overall gene–trait associations. When applied to COVID-19 GWAS summary data, SUMMIT identifies risk genes for COVID-19 severity. However, the predominance of European ancestry in most eQTL data necessitates the corresponding eQTL summary data for extension to other ancestral populations.

Existing TWAS methods focus primarily on examining individual outcome traits separately in a univariate manner. However, given the shared genetic basis among numerous human complex traits, the potential for increased effectiveness of TWAS methods through multivariate analysis cannot be ignored [93,94].

To address this issue, moPMR-Egger [95] defines instrumental variables as possible LD between *cis*-SNPs of a gene and investigates its causal effect on multiple traits simultaneously. The significant aspect of moPMR-Egger is its capacity to evaluate and control for horizontal pleiotropic effects resulting from instruments, thereby increasing power while reducing false associations for TWAS methods.

Enhancers are crucial DNA regulatory elements that regulate the expression of target genes, and can be transcribed with expression levels as a signal for their activation [96,97]. Consequently, previous eQTL analysis may have missed essential information about enhancer-mediated genetic mechanisms. A recent population-scale analysis of enhancer expression in the human cerebral cortex [98], based on the integration of cell type-specific transcriptome and regulome data, revealed that enhancer eQTLs and genes account for a significant percentage of the heritability of neuropsychiatric traits. Enhancer eQTLs improved the functional interpretation of trait–locus associations in TWAS.

In addition to enhancers, TFs are essential regulatory elements for gene expression [99,100]. To fill this gap, sTF-TWAS [101,102] was developed to integrate prior knowledge of susceptible TF (sTF)-occupied *cis*-regulatory elements (STFCREs) with TWAS. This approach predicts gene expression using putative genetic variants located in STFCREs and provides evidence that genetic variants mediate the binding affinity of susceptible TFs to impact disease risk.

Current TWAS methods mainly focus on one gene at a time, while complex diseases typically result from biological networks involving multiple genes. From this perspective, PoLoNet [103] was developed to identify the association between specific networks and binary or ordinal categorical traits. PoLoNet is applied in the traditional two-stage TWAS framework. First, it obtains the SNP effect on genes using a distribution-robust nonparametric DPR model. Then, pointwise mutual information (PMI) is used to calculate all the between-node or edge correlations of predicted gene expression, followed by a proportional odds logistic model to perform association analysis on all nodes and edges. The biological network is selected from pathways in the Kyoto Encyclopedia of Genes and Genomes (KEGG), ultimately identifying the trait-related network nodes or edges.

#### Extension in training with summary statistics

In the first step of traditional TWAS frameworks, numerous methods estimate the effect size of SNPs using individual-level reference expression and genotype data. However, utilizing individual-level data in TWAS is more restricted compared to GWAS summary statistics, which have a broader range of applications. This is because GWAS summary statistics typically have larger sample size and are more readily accessible. TWAS is analogous to polygenic risk score (PRS) [104,105], which includes several methods using GWAS summary statistics instead of individual-level data. Therefore, it is feasible for TWAS to use summary-level data to indicate molecular mechanisms of genetic variations by integrating eQTL data and GWAS summary statistics.

For instance, OTTERS [106] is a TWAS framework that synthesizes various PRS methods [107–109] to estimate eQTL effect sizes from eQTL summary statistics, similar to GWAS summary statistics. OTTERS can successfully identify potential risk genes that may be missed using limited individual-level datasets.

A previous study compared schizophrenia TWAS results based on datasets at two different levels [110]. The results showed that the model using summary statistics outperformed the model using individual-level data for many genes. For schizophrenia, several new susceptibility genes were identified that were not noticeable in other models. Thus, using GWAS summary statistics in TWAS reveals an effective way to elucidate the mechanisms of genetic variations and expands the utility of TWAS. Particularly, integrating GWAS summary statistics with eQTL data provides a feasible approach to understanding complex phenotypes.

#### Identification of susceptibility genes based on TWAS

Recently, a comprehensive compilation of TWAS discoveries named TWAS Atlas [111] was presented, offering high-quality human gene–trait associations gleaned from 200 peer-reviewed papers. Here we manually curated the publications of gene–disease association studies based on TWAS methods from the TWAS Atlas database, as well as the papers that were omitted in the TWAS Atlas.

First, we conducted a targeted exploration of the disease types and collected 347 entries from 168 papers by using the “browse” page of the TWAS Atlas website (https://ngdc.cncb.ac.cn/twas/browse). Subsequently, we performed an exhaustive literature search on the PubMed database using the keywords “transcriptome-wide association study” and “disease”, meticulously evaluating results to supplement any gaps in the TWAS Atlas. In doing so, we procured 131 entries from 67 distinguished papers. For better classification of diseases, we converted all annotation properties of each disease from the Experimental Factor Ontology (EFO) [112] to MeSH (https://www.ncbi.nlm.nih.gov/mesh) terms (Table S1).

**Figure 3:**
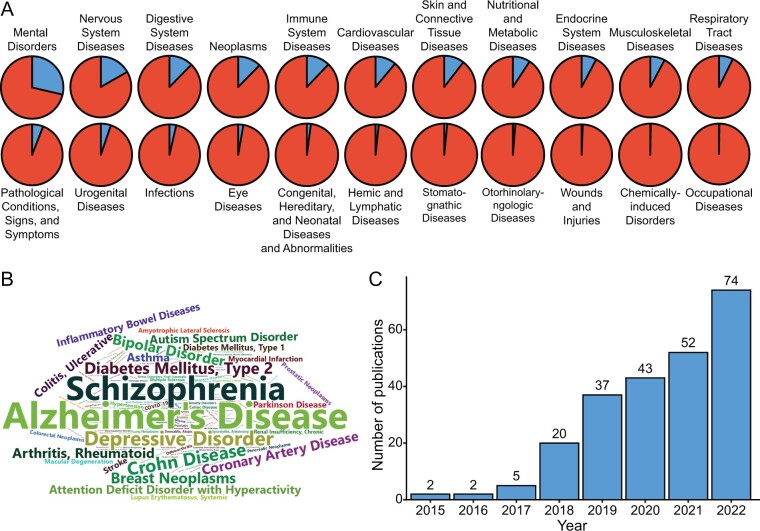
Statistical results of disease types and publications of identifying susceptibility genes based on TWAS methods **A**. Pie chart showing the proportion of each MeSH disease category in the gene–disease association publications based on TWAS. Blue denotes the portion attributed to each disease, while red represents the all disease background. **B**. A word cloud graph showing all disease terms. Font size is determined by the frequency of each disease. **C**. Bar chart showing the number of publications that conduct gene–disease association analyses based on TWAS each year.

Various pie charts were created to demonstrate the distribution of different disease categories among the 478 entries (**Figure 3**A). The R package “wordcloud2” was utilized to construct wordcloud images, which were used for analyzing disease frequency (Figure 3B). The bar chart (Figure 3C) illustrates the number of publications per year, with 235 publications in total.

Out of the 22 disease categories identified, “Mental Disorders” was the most prevalent, accounting for 29% (137/478) of all cases. The top 3 diseases among the total 149 diseases were “Alzheimer’s Disease” (35/235), “Schizophrenia” (31/235), and “Depressive Disorder” (19/235) (Figure 3B). TWAS has also been employed to investigate complex traits in numerous species including plants, animals, and humans, which highlights its powerful statistical performance in fully identifying the genetic mechanisms underlying these traits.

AD is a prevalent form of dementia in older individuals, initially manifesting as memory loss and progressing to severe executive and cognitive impairment [113]. Current therapies only manage symptoms without curing the disease [114], resulting in significant economic and societal burdens. Consequently, effective risk assessment procedures for AD are essential. Despite the development of numerous risk assessment strategies, inconsistencies across studies have been noted, possibly due to limitations like small sample size, selection bias, potential confusion, and reverse causality.

Various TWAS methods have been utilized in AD research. For instance, a previous study using FUSION identified four novel AD-associated genes (*MLH3*, *FNBP4*, *CEACAM19*, and *CLPTM1*) in brain tissue, adipose tissue, and whole blood [115]. Another study integrated expression and intron usage into the FUSION framework to investigate the association of alternative splicing with AD, uncovering 21 genes significantly linked to AD at the gene expression or intronic excision level [116]. Through the TWAS statistical framework UTMOST, 69 AD-associated genes were pinpointed by analyzing 17,008 cases and 37,154 controls [45]. Additionally, research employing PrediXcan to identify susceptibility genes associated with AD discovered 50 distinct genes and 126 tissue-specific correlations [117].

While numerous TWAS studies have explored AD, results often exhibit variability due to lack of a reliable gold standard to evaluate results, and not all identified genes and associations are necessarily causal with disease. Furthermore, heterogeneity among sample batches and factors like sample size, tissue origin, and ancestor population may influence final results. Hence, selecting appropriate analysis tools based on distinct sample data and analytical requirements is particularly crucial (Table S2).

### EWAS

The concept of epigenome-wide association study (EWAS) was first introduced in 2011 [118] to explore epigenetic features linked to the susceptibility and progression of complex human diseases, similar to GWAS which connects the epigenome to phenotype through investigating modifications such as methylation across various disease states. Currently, EWAS analysis primarily centers on methylomes, often utilizing microarray-based methylation data due to the cost of whole-genome bisulfite sequencing (WGBS) [119].

EWAS analysis can be categorized into two main approaches [119]: differentially methylated positions (DMPs) and differentially methylated regions (DMRs). DMP analysis correlates methylation levels of individual CpG sites (using beta- or M values) with the phenotype, while DMR analysis links genomic regions containing multiple adjacent DMPs to the phenotype. Various tools exist for DMP analysis. For example, in case-control studies, the R package CHAMP [120] identifies significant differential CpG sites based on mean methylation levels from a beta-matrix after preprocessing raw microarray data through probe filtering, imputation, and normalization.

Additionally, the EWAS2.0 software [121] was developed for EWAS analysis within the “population epigenetic framework”. Like GWAS analysis, EWAS2.0 can conduct epigenome-wide single-marker association studies, methylation haplotype association studies, and association meta-analyses using chi-square tests, *t*-tests, linear regression, and logistic regression. Before analysis, methylation data inputted into EWAS2.0 should undergo preprocessing and normalization similar to those in CHAMP [119].

In response to the improvement of EWAS and the growing availability of microarray-based data, EWASdb [122] curated 1319 EWASs associated with 302 phenotypes, including EWASs for single markers, KEGG pathways, and GO (Gene Ontology) categories. Recently, the EWAS Open Platform [123] was developed and comprises the EWAS Atlas [124], EWAS Data Hub [125], and EWAS Toolkit [123]. The EWAS Atlas [124] has curated 675,261 epigenome–phenotype associations from 1079 publications related to 798 traits. The EWAS Data Hub [125] provides normalized DNA methylation array data from 143,678 samples across 1210 tissues/cells associated with 658 diseases. The EWAS Open Platform [122] offers comprehensive resources, data, and tools for EWAS enrichment, annotation, and visualization.

### PWAS

Following TWAS, PWAS [16] was introduced in 2020, based on the assumption that genetic variants within coding regions can influence phenotypes by affecting the biochemical functions of protein products. This novel framework identifies gene–trait associations mediated by protein functional changes. It estimates protein damage based on an individual’s genotype to capture the impacts of variations that affect genes’ coding regions, such as missense, nonsense, and frameshift. PWAS initially computes effect scores for non-synonymous variants within each gene’s coding region, indicating their potential to disrupt the gene’s protein product. These scores range from 0 (indicating complete loss of function) to 1 (suggesting no functional impact). Missense, nonsense, frameshift, in-frame insertion and deletion (indel), and canonical splice-site variants are recognized as influencing protein function. FIRM [34], a machine learning model that considers the rich proteomic context of each impacting variant, is employed to calculate the effect scores for missense variants, while nonsense, frameshift, and canonical splice-site variants are categorized as loss-of-function and assigned a score of 0. Regarding in-frame indels, the effect score is determined based on the number of altered amino acids.

By combining genotyping information from the cohort with these predicted effects, PWAS generates functional prediction of each gene, representing each protein-coding gene’s functional effect score. Statistical analysis is then performed to determine whether a gene’s effect score is associated with phenotype. A significant association between the effect scores of cases and controls, in the case of a binary phenotype, would indicate that the protein is more (or less) damaged in affected individuals. Simulation results show that PWAS performs exceptionally well in cases of recessive inheritance, and its discovery power is robust when applied to real datasets. Additionally, PWAS identified numerous known associations for most phenotypes. For instance, when the PWAS was applied to the UKBB cohort [126,127] to assess its applicability to different phenotypes, 5249 of the 500,000 participants contained a GWAS-significant non-synonymous variant in the gene’s coding region.

One of PWAS’s distinctive characteristics is its ability to model both dominant and recessive inheritance. While substantial evidence suggests that the commonly used additive model can capture most of human trait heritability, non-additive and epistatic effects are crucial in many phenotypes [128]. However, PWAS’s reliance on complete individual-level data, including genotype and phenotype, could be a drawback, preventing it from examining GWAS summary statistics alone, unlike other approaches. This dependency on raw data is due to the non-linear aggregation algorithm used to obtain gene impact scores from variation effect scores.

### CWAS

GWAS and TWAS have identified numerous risk-associated genetic variants, but the mechanisms of non-coding genetic variants that influence complex traits and diseases remain to be fully explored. Many studies have shown that eQTLs influence gene expression by altering chromatin activity, which has led to increased research attention on the impact of risk-associated genetic variants on chromatin [129–135].

Analogous to eQTL, cQTL is a SNP associated with chromatin states, such as histone modifications, TF binding, and chromatin accessibility. Moreover, allelic imbalance in epigenomic data, defined as differences in the representation of heterozygous SNP alleles in sequencing reads, can be employed to identify variations that influence chromatin states [135–138].

Nevertheless, the application of cQTL and allelic imbalance to understand trait heritability is limited by two main factors: first, the lack of substantial reference epigenomes from relevant organs, and second, the absence of a uniform methodology for incorporating these data into GWAS. To overcome these limitations, a novel approach named CWAS [17] has been introduced. CWAS aims to identify genetic determinants of TF binding and histone modifications, and associates genetically predicted chromatin signals with traits based on GWAS summary statistics.

To implement CWAS, the growing number of chromatin immunoprecipitation followed by sequencing (ChIP-seq) datasets was leveraged to create and benchmark an approach that imputes genotypes from ChIP-seq data with high accuracy. As an extension of the conventional TWAS pipeline, CWAS takes into account allele-specific information and chromatin phenotype.

CWAS findings demonstrate the efficacy of an integrative cistrome approach in identifying genetic determinants of gene regulation. Furthermore, CWAS is shown to complement other methods, such as TWAS/eQTL-based methods, which may fail to detect associations involving genes with intricate regulation and context-dependent expression. In the context of prostate cancer, CWAS identifies key regulatory elements and androgen receptor-binding sites, explaining the associations at 52 out of 98 known prostate cancer risk loci. Additionally, CWAS discovered 17 novel risk loci, emphasizing the power of this approach in uncovering previously unidentified genetic determinants of disease risk.

### RWAS

Researchers have developed an innovative work, known as RWAS [21], which utilizes cancer ATAC-seq datasets from The Cancer Genome Atlas (TCGA) to identify germline as-aQTLs and links them to potential risk mechanisms. ATAC-seq is a sequencing-based technique that enables the identification of accessible regions within the genome, often correlated with TF-binding sites [135,139]. RWAS has successfully been applied to seven cancer GWAS datasets, revealing numerous cancer-specific as-aQTLs that demonstrate a higher enrichment for cancer risk heritability compared to other functional annotations. Moreover, the majority of these cancer-specific as-aQTLs have been observed to modulate TF patterns, thereby influencing differential TF binding and gene expression. RWAS has identified genetically linked accessible peaks in over 70% of recognized breast and prostate loci and has unearthed novel risk loci across all cancer types analyzed. Through the integration of as-aQTL discovery, motif analysis, and RWAS, potential susceptibility regulatory elements and their likely upstream regulators have been identified.

Another concurrent study also referred to as RWAS [22], aims to pinpoint particular enhancer attributes potentially contributing to genetic disease risk. The RWAS methodology comprises three core stages. First, genotyped SNPs are mapped to regulatory features like enhancers specific to distinct cell types or tissues. Subsequently, the association between each regulatory feature and a trait of interest is examined. Finally, enhancer-set enrichment analyses are performed to disclose quantitative or categorical features of regulatory elements associated with the trait. These procedures denote a novel application of MAGMA [140], originally designed for gene-centric GWAS analysis.

### IWAS

IWAS was initially proposed in 2017 [18] to investigate the relationship between genes and complex diseases via imaging endophenotypes. The protocol of IWAS is analogous to TWAS. The difference is that IWAS substitutes gene expression with imaging endophenotypes in the Alzheimer’s Disease Neuroimaging Initiative (ADNI) database. Analogous to TWAS, the elastic net was employed to train the weights of genetic variations for each imaging endophenotype and subsequently compared with gene expression-based weights from PrediXcan. Distinct tests were conducted utilizing sum of powered score (SPU) (1), SPU (2), and aSPU for each set of weights, respectively, with numerous significant associations using imaging-based weights while non-significant associations using expression-based weights. Moreover, a test integrating multiple weights named doubly aSPU (daSPU) test was applied to imaging endophenotypes, unveiling a substantial number of significant genes. Conversely, a small number of significant genes overlapped with GWAS were detected when applied to the International Genomics of Alzheimer’s Project (IGAP) GWAS summary statistics. These results demonstrate that univariate IWAS (UV-IWAS) has higher power in specific complex diseases (*e.g.*, AD) and daSPU-based IWAS has a better interpretation through the imaging endophenotypes.

Subsequently, multivariate IWAS (MV-IWAS) [19] was introduced as an extension of UV-IWAS to reduce type-I errors originating from horizontal pleiotropy, with the residual pleiotropic effects addressed via Egger estimators denoted as MV-IWAS-Egger. Under the scenario of directional pleiotropy, MV-IWAS-Egger controls the type-I error well and has maximum power across all multivariate models. Notably, causal associations for the left hippocampus and right inferior temporal cortex volumes concerning AD were pinpointed when both tests were applied to the ADNI endophenotypes, a conclusion supported by extensive literature. Furthermore, MV-IWAS identified numerous new causal brain phenotypes based on UKBB data which were missed by UV-IWAS, and also implicated many potential false-positive UV-IWAS results.

Recently, a preprint named BrainXcan [20] was published, which utilizes GWAS data to test the associations between genetic predictors of brain magnetic resonance imaging (MRI)-derived features and complex traits based on three modules as an extension of PrediXcan. First, the “Prediction weight training” module computes image-derived phenotype (IDP) prediction weights, IDP QTLs, and “reference LD” information across the UKBB and Psychiatric Genomics Consortium (PGC) data. Then, the “Association” module will generate regression estimated coefficients between predicted IDPs and traits. Last, the “Mendelian randomization” module examines the direction of causal flow, *i.e.*, whether phenotype influences disease or disease affects phenotype. BrainXcan identified many risk phenotypes supporting the disconnectivity hypothesis of schizophrenia.

In conclusion, IWAS achieved enhanced power and interpretability through the integration of imaging datasets. However, limitations persist, including the size of the training cohort constraining the predictor’s efficacy. Further investigation is required to validate the causal relationship, and the enhancements to the linear regression model are necessary.

### mGWAS and MWAS

Metabolomics-based genome-wide association study (mGWAS) links thousands of metabolites and millions of genetic variants to understand the genetic regulation of metabolites in complex phenotypes, crucial for unraveling their genetic underpinnings [141,142]. mGWAS involves examining genetic variants across the entire genome to identify associations with metabolite-level variations, typically utilizing large-scale datasets containing genotypic and metabolomic data. Predominantly conducted in populations of European ancestry, these studies predominantly focus on blood and urine metabolites, employing proton nuclear magnetic resonance (NMR) spectroscopy or mass spectrometry (MS) for quantification [143]. By elucidating the genetic basis of metabolite traits, mGWAS offers insights into metabolic pathways and their regulation, aiding in identifying disease biomarkers, understanding metabolic dysregulation, and discovering therapeutic targets [144].

Metagenome-wide association studies (MWAS) are primarily modeled on the GWAS framework, aiming to identify genes in the human metagenome associated with phenotypes, often diseases. In MWAS, gene relative abundance in a metagenome is correlated with a disease of interest, typically after grouping genes into strain-level clusters termed metagenomic linkage groups, metagenomic clusters, or metagenomic species to reduce data dimensionality [145]. MWAS entails analyzing the genetic composition of microbial communities (the metagenome) through sequencing microbial DNA from samples like fecal, oral, or skin swabs, followed by bioinformatic analysis to characterize microbial taxa or functional genes [146]. By identifying microbial taxa or functional genes associated with specific phenotypes or diseases, MWAS sheds light on host-microbiome interactions, microbial community dynamics, and the impact of environmental factors on the microbiota [147,148].

In summary, mGWAS and MWAS are robust methodologies enabling researchers to investigate the genetic basis of metabolite concentrations and microbial community composition, respectively. These approaches offer crucial insights into the intricate interplay between genetic and environmental factors in health and disease, with implications for personalized medicine, biomarker discovery, and therapeutic interventions.

## Discussion

Multiome-wide association study methods offer an effective strategy for integrating diverse multiomic data, addressing the challenge of interpreting GWAS significance signals within non-coding regions to some extent and enhancing the statistical power of genome–phenotype associations. These studies aim to explore the impact of various factors, including genomes, epigenomes, transcriptomes, proteomes, metabolome, and microbiomes, on phenotypes, and their interactions within biological systems. Compared to single-omics research, multiomics integration provides a more comprehensive approach, considering the complexity and diversity of human traits and diseases.

Recent research shows a tendency toward joint analysis by integrating GWAS with multiome-mediated methods. For instance, an integrated analysis of GWAS and TWAS on patients with drug-induced liver injury successfully identified significant clinical risk predictors, aiding in the susceptibility assessment to liver injury due to amoxicillin-clavulanate [149]. Another study conducted an integrated analysis of GWAS and TWAS on type 2 diabetes mellitus and schizophrenia to explore shared pathways between the two diseases [150]. Similarly, an integrated analysis of TWAS, PWAS, Bayesian colocalization, and MR on Parkinson’s disease prioritized two susceptibility genes based on their effects on brain proteins and transcriptome levels [151]. These studies underscore the potential of integrated analyses of multiome-mediated methods in understanding human complex diseases.

The findings of research through multiome-mediated methods have proven to be significant reference values in investigating complex traits and diseases. However, several limitations persist in multiome-mediated methods. Firstly, while many significant signals identified by these approaches are associated with diseases, they may not necessarily be causal. Moreover, these methods typically rely on paired public genotypic and multiomic data as reference panels, which are still scarce in large population cohorts, often restricted by privacy concerns, and particularly lacking in most tissues, especially among populations of non-European ancestries. Additionally, comprehensive data resources for multiome-mediated methods remain relatively limited. Currently, only three databases for TWAS (TWAS-hub [152], webTWAS [86], and TWAS Atlas [111]), and four databases for EWAS (EWASdb [122], EWAS Atlas [124], EWAS Data Hub [125], and EWAS Open Platform [123]) are available. Lastly, the rapid advancement of single-cell sequencing has underscored the importance of analyzing disease mechanisms from a single-cell perspective, emphasizing the need to integrate single-cell data into multiome-wide association study methods.

In summary, future studies will be required to distinguish causal signals of complex human diseases or traits and elucidate single cell-specific genetic mechanisms. With the release of large datasets such as UKBB, multiome-wide association study methods are poised to experience an influx of new method extensions.

## CRediT author statement

**Mengting Shao:** Data curation, Visualization, Writing – original draft, Writing – review & editing. **Kaiyang Chen:** Writing – original draft. **Shuting Zhang:** Writing – review & editing. **Min Tian:** Writing – original draft. **Yan Shen:** Writing – review & editing. **Chen Cao:** Conceptualization, Supervision, Writing – review & editing, Project administration. **Ning Gu:** Conceptualization, Supervision. All authors have read and approved the final manuscript.

## Supplementary Material

qzae077_Supplementary_Data
