# Supplementary material for: Multiome-wide Association Studies: Novel Approaches for Understanding Diseases
Source: Genomics Proteomics Bioinformatics. 2024 Oct 29;22(5):qzae077. doi: 10.1093/gpbjnl/qzae077 (PMC11630051; doi:10.1093/gpbjnl/qzae077)
Supplement: qzae077_Supplementary_Data [file qzae077_supplementary_data.zip › supplementary material captions.docx]

**Supplementary material**

**Table S1 A detailed list of each curated entry, including disease category and publications**

**Table S2 Comparison of the performance of various algorithms across different studies**

**Table S3 Abbreviation table**
